# Cloning Approaches for Identifying Aging and Longevity-Related Genes in Mammals

**DOI:** 10.1100/tsw.2002.117

**Published:** 2002-02-22

**Authors:** Davina C. Simoes, Efstathios S. Gonos

**Affiliations:** National Hellenic Research Foundation, Institute of Biological Research and Biotechnology, 48 Vas. Constantinou Ave., Athens 11635, Greece

**Keywords:** aging, longevity, senescence, subtractive hybridization, differential display PCR, microarray, gene

## Abstract

Aging is a phenomenon that affects nearly all animal species. Several studies using different systems have identified a number of processes thought to contribute to the aging phenotype. Many differentially expressed genes have been implicated, but the mechanisms governing mammalian aging (and longevity) are not yet fully understood, and the list of concerned genes is still incomplete and fragmented. Different approaches have been used to clone aging and longevity-related genes. In this article we review these cloning techniques and discuss their advantages and limitations. Further research on the function of these genes as well as the network of their protein products will give better insight into the aging process as a whole and its associated pathologies.

## INTRODUCTION

Aging is a phenomenon that affects nearly all animal species. It is usually defined as the progressive loss of function accompanied by decreasing fertility and increasing mortality with advanced age. It can be considered as the product of interactions between genetic, environmental, and lifestyle factors, which in turn influences longevity that varies between and within species. The question of why we age has intrigued mankind since the beginning of time and still its molecular basis remains unknown. Several studies using different systems including yeast, *Caenorhabditis elegans,* Drosophila, and mice, as well as human cells and tissues, have identified a number of processes thought to contribute to the aging phenotype[1]. Postulated mechanisms include accumulative DNA damage leading to genomic instability, epigenetic alterations that lead to altered gene expression patterns, telomere shortening in replicative cells, oxidative damage to critical macromolecules by reactive oxygen species (ROS), and nonenzymatic glycation of long-lived proteins[1,2]. To understand the molecular regulation of these processes, the relevant subsets of differentially expressed genes of interest must be cloned, identified, and studied in detail. Even though we are entering the postgenomic era and a substantial list of genes has emerged that associate with various models of aging, the list of genes and pathways involved in aging phenomenon is still fragmented and incomplete. This minireview article will describe the cloning approaches used to screen for changes in gene expression in mammalian aging systems, giving emphasis to their limitations, advantages, and disadvantages.

## THE USE OF REPLICATIVE SENESCENCE AS A MAMMALIAN *IN VITRO* AGING SYSTEM

Normal mammalian cells cultured *in vitro* undergo a finite number of divisions before entering a stable and irreversible state of growth arrest; a process known as replicative senescence[3]. This model has been used widely to study *in vitro* aging. For instance, it has been employed in both embryonic and adult cells (usually biopsies), as well as in various cell types such as fibroblasts, osteoblasts, and keratinocytes. In the early 1980s it was observed that there is almost a linear association between species maximum lifespan and their corresponding fibroblast-derived cultures *in vitro* lifespan[4]. These findings support the hypothesis that replicative senescence is the manifestation of aging *in vitro*, and fibroblasts derived from patients suffering with progeroid syndromes, such as Werner's Syndrome (WS), undergo fewer populations doublings than normal fibroblasts[5]. However a laborious study by Cristofalo et al.[6] has shown more recently in humans that there is a lack of association between donor's age and their skin fibroblasts *in vitro* lifespan. Therefore, although replicative senescence remains the most powerful and popular *in vitro* system for studying aging in mammalian cells, one should bear in mind its limitations as far as its direct analogy to *in vivo* aging is concerned.

## PLUS/MINUS DIFFERENTIAL SCREENING AND SUBTRACTIVE HYBRIDIZATION

The first differential screening method to be used for cloning age-related genes, the so-called plus/minus screening, is based in the direct comparison of the hybridization profile of two replica filters from a given cDNA library of two different probes (for example, one derived from proliferating cells and the other from senescent cultures). Lecka-Czernik et al.[7] were the first to use such a technique to clone differentially expressed genes from WS fibroblasts. They identified 15 cDNA clones that were overexpressed in both senescent and WS human diploid fibroblast (HDF) encoding among others for fibronectin, SPARC, α2-chimerin, EF-1α, elF-2β, and acid sphingomyelinase as well as for few novel sequences.

Because plus/minus differential screening is restricted to abundantly expressed genes, subtractive cDNA hybridization has been developed as a powerful approach to identify and isolate cDNAs corresponding in more rarely expressed genes[8,9]. Numerous cDNA subtraction methods have been reported. In general, they involve hybridization of cDNA from one population (tester) to excess of mRNA (cDNA) from another population (driver) and then separation of the unhybridized fraction (target) from the hybridized common sequences. The latter step is usually accomplished by hydroxypatite chromatography[10], avidin-biotin binding[8], or oligo$(dT)_{30}$-latex beads[9]. These subtraction techniques often require greater then 20 μg of poly$(A)^+$ RNA, involve multiple or repeated subtraction steps, and are labor intensive, but as mentioned earlier are suited for the identification of rare messages. The needed high amounts of poly$(A)^+$ RNA essentially restrict these analyses to samples that can be cultured and grown *in vitro*.

We have applied a combination of both of these cloning approaches to clone senescence-related genes. For these purposes, a clonal system of conditional SV40 T antigen rat embryo fibroblast immortalized cell lines, which undergo senescence upon T antigen inactivation, has been used. These conditional cell lines, the tsa cell lines, have an unlimited proliferative capacity at 33˚C, but become irreversibly growth arrested and undergo senescence upon shift up to 39.5˚C, the temperature at which the tsA58 T antigen is rapidly degraded[11]. Construction of cDNA libraries from the two conditional cell lines and application of both plus/minus differential screening and subtractive hybridization techniques have resulted in the cloning of eight senescence-induced genes (SGP-2/Apo J, α1-procollagen, osteonectin, fibronectin, SM22, cytochrome C oxidase, GTP-α like binding protein and a novel gene) and a senescence-repressed gene (FRS-2)[12]. RNA analysis has shown that the senescence-induced genes are overexpressed in senescent human and rodent fibroblasts and osteoblasts, as well as in fibroblasts undergoing premature senescence due to exposure to various stress-inducing agents[11,13] (Petropoulou et al., submitted). Finally, for at least one cloned gene, it has been shown that its expression also relates to *in vivo* aging[14].

An optimized subtraction cloning hybridization method was developed more recently by Diatchenko et al.[15]. The method is termed suppression subtractive hybridization (SSH) and describes a PCR-based cDNA subtraction. This method aims to overcome the problem of differences in mRNA abundance and claims to be efficient to obtain low-abundance transcripts. Based on this technique, Clontech (Palo Alto, CA) has developed the PCR-SelectTM cDNA Subtraction kit that requires only 0.5–2 μg of poly(A)$^+$ RNA and, in addition, is much more efficient and simplified, compared to traditional methods. This method enables researchers to compare two populations of mRNA as follows. First both mRNA populations are converted into cDNAs, and amplification of sequences of identical abundance in the two populations is suppressed. Tester and driver double-stranded cDNA are prepared from mRNA samples to be compared. Then the samples are digested with a restriction endonuclease that results in blunt-ended molecules. Two tester populations are created with different adaptors but the driver cDNA has no adaptor. Hybridization kinetics leads to equalization and enrichment of differentially expressed sequences. Using suppressing PCR, only differentially expressed sequences are amplified exponentially. Background is reduced and differentially expressed sequences are further enriched (Fig. 1). Therefore, SSH is used to selectively amplify target cDNA fragments (which are differentially expressed) and simultaneously suppress nontarget DNA amplification.

Grillari et al.[16] have applied SSH to clone senescence-related genes from endothelial cells. Their study has identified a total of 17 genes in which 12 were mRNA with increased levels in the senescent cells (β-IG-H3, SEC13R, PIG3, fibronectin, L6/TAXREB 107, IGFBP-3, PAI-I, Staf50, NSPL-1, ribosomal protein L28, and two unknown genes) and five were mRNA with increased levels in young cells (LDH-B, RDC-1, TF II S, and two unknown genes).

## DIFFERENTIAL DISPLAY PCR

With the advent of PCR in the early 1990s, a new technique called Differential Display PCR (DD-PCR) was developed enabling researchers to compare and visualize the difference in gene expression in individual samples[17]. The mRNA DD-PCR technology works by systematic amplification of the 3’ terminal portions of mRNA and resolution of those fragments on a DNA sequencing gel. Using anchored primers designed to bind the 5’ boundary of the poly-A tails, followed by PCR amplification with additional upstream primers of arbitrary sequences, mRNA subpopulations are visualized by denaturing polyacrylamide electrophoresis (Fig. 2). This allows direct side-by-side comparison of most of the mRNA between or among related cells. The DD-PCR method is thus far unique in its potential to visualize most of the expressed genes in a given cell in a systematic and sequence-dependent manner by using multiple primer combinations. The method also enables the recovery of sequence information and the development of probes to isolate their cDNA and genomic DNA for further molecular and functional characterizations. The major advantage of such an approach compared with subtractive cDNA hybridization is that DD-PCR has the ability to compare more than two RNA samples at once and uses very small amounts

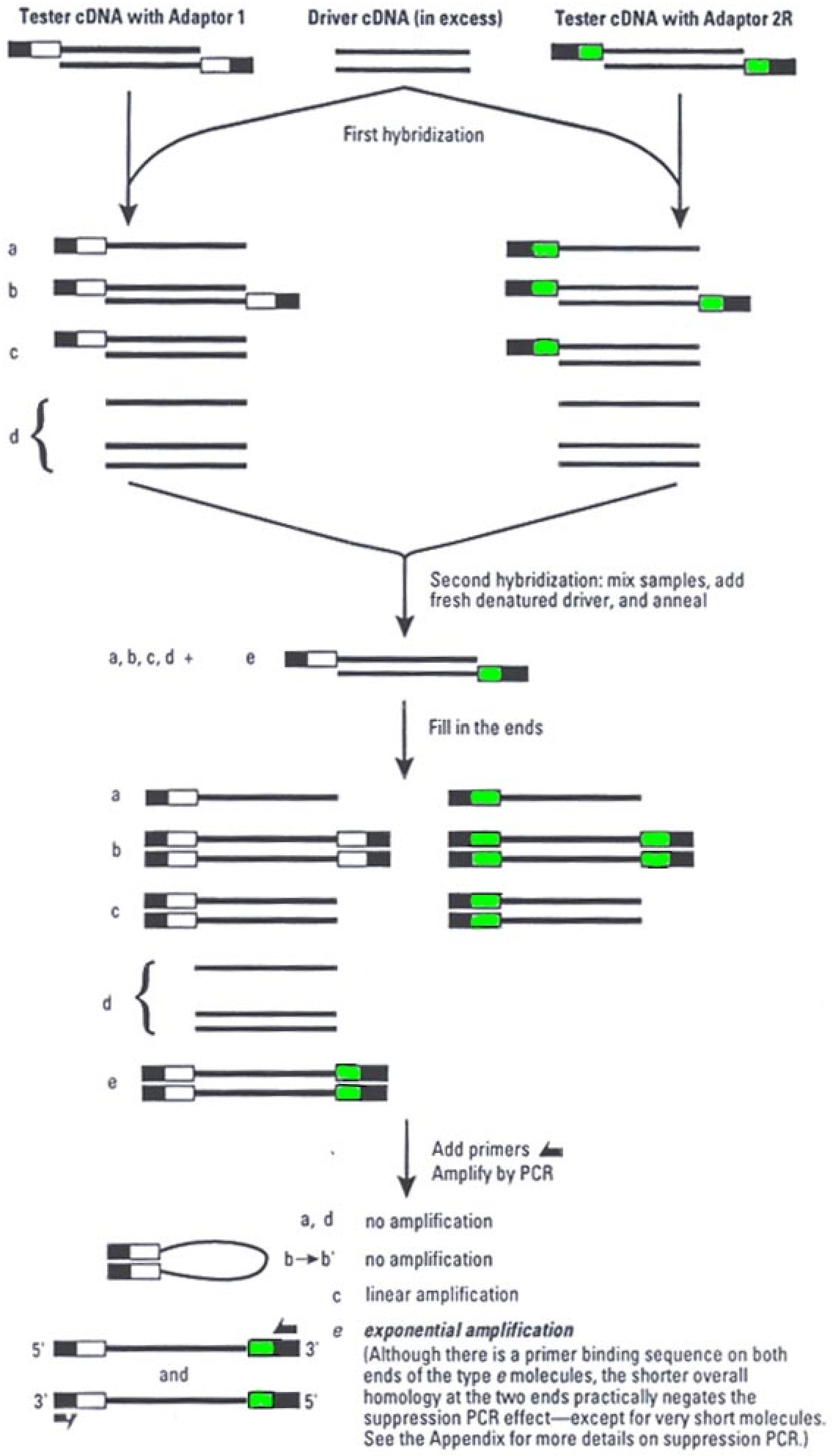

**FIGURE 1.** Schematic diagram of the SSH. Type ***e*** molecules are formed only if the sequence is up-regulated in the tester cDNA. Solid lines represent the Rsa I-digested tester of driver cDNA. Solid boxes represent the outer part of the Adaptor 1 and 2R longer strands and corresponding PCR primer 1 sequence. Green boxes represent the inner part of the Adaptor 1 and the corresponding PCR primer 1 sequence. Green boxes represent the inner part of Adaptor 2R and the corresponding nested PCR primer 2R sequence. (Figure provided by BD Biosciences CLONTECH.)

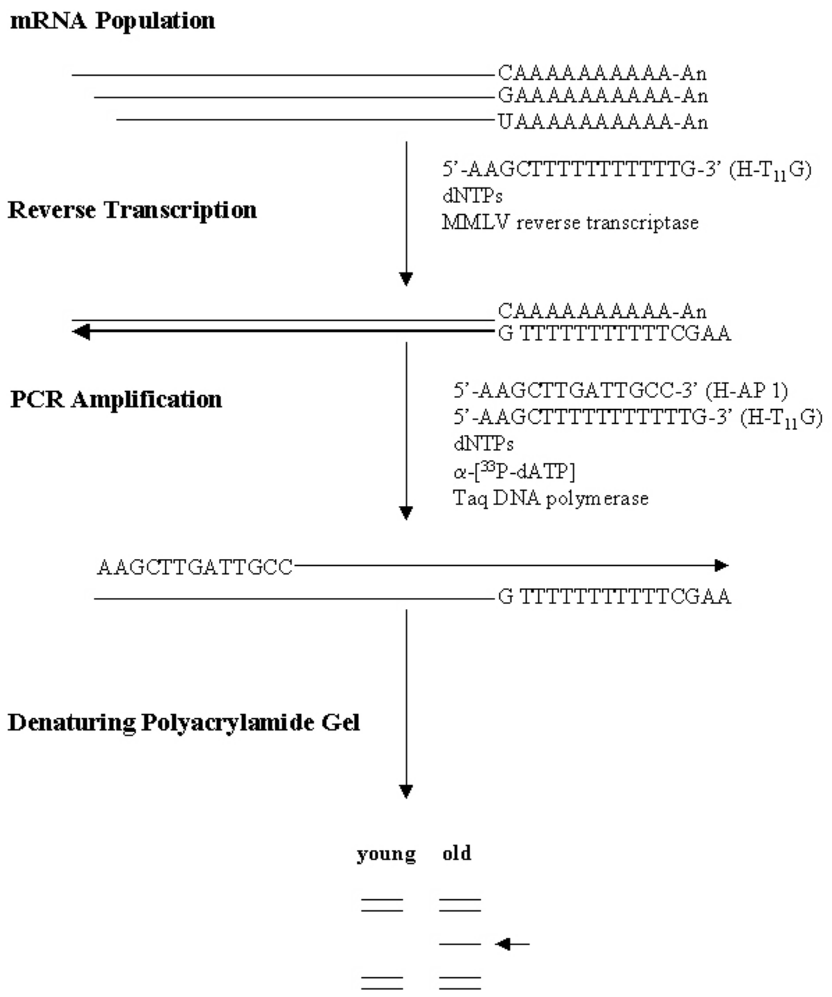

**FIGURE 2.** Schematic diagram of the DD-PCR.

of total RNA (0.2 μg) instead of poly(A)$^+$ RNA thus enabling the direct analysis from tissues and biopsies and avoiding the need of culturing cells *in vitro*[18].

In past years DD-PCR was cited extensively in the literature and resulted in several hundred publications reporting its successful application in different areas of research. However, the DD-PCR technique was found to contain many more stumbling blocks than could be guessed. The main disadvantage is the very well-known false positives of the technique, which lead the researcher to carry on innumerable Southern and Northern blots. Many variations of the technique were developed to overcome this disadvantage[19,20,21,22,23,24].

Linskens et al.[21] describe a catalogue of genes with altered expression in *in vitro* senescent cells using a modified DD-PCR method. A total of 23 genes (12 known, 11 novel) were identified that include 80K-L, ALDH-1, Laminin A, COL3α1, COL1α1, PAI-2, HTF, IFN-γ, CD44, IGFBP-5, SOD, and Heregulin. The main difference from the original technique was the introduction of a two-step PCR amplification, in which the first four PCR cycles were done at low annealing temperature, while efficient amplification was carried out in subsequent PCR cycles at a higher annealing temperature.

Further improvements in the technique were used by aiming to compare differences in gene expression levels in the brains, livers, and kidneys of young and old rats[18]. The higher sensitivity of the technique was achieved by (1) increasing the size of the PCR primers, (2) incorporation of a HindIII site at their 5' ends, (3) use of one-base instead of two-base anchored primer[25], (4) use of $^{33}$P-labelled nucleotides instead of $^{35}$S-labelled nucleotides[25], and (5) use of a Beckman Genomyx DNA sequencing rig[20].

In our laboratory we overcame the problem of false positives by adding two screening steps of single-strand conformation polymorphism (SSCP) by the use of RNA image kit (GenHunter, Nashville, TN). We observed that the main sources of false positives are due to (1) identical-sized DNA fragments that comigrate with the band of interest on display gels and (2) DNA contamination introduced into the reamplification PCR. Even under ideal conditions, after extracting the cDNA from the sequencing gel, several comigrating cDNA species may be present together with the cDNA of interest. Although originally present as only a very small proportion of the total DNA recovered, after the standard 40 cycles of PCR, these comigrating species will be amplified to equivalent levels of the real candidate cDNA. In that case, after cloning, a very laborious series of Northern blots had to be applied to separate the clones containing the cDNA of interest. SSCP is an electrophoresis protocol designed to separate single-stranded DNA fragments on the basis of conformation rather than size. Therefore, SSCP will separate individual strands of a DNA duplex. After cutting and extracting the DD-PCR cDNA band of interest from a denaturing polyacrylamide gel, we reamplified the PCR for only ten cycles and subsequently ran it on an SSCP gel. Visualization of SSCP gel by autoradiography has permitted us to reconfirm patterns of expression observed on display gels for all DD-PCR. The band of interest was cut from the SSCP gel, and after another round of ten cycles of amplification, the cDNA was cloned. As shown in Fig. 3, ten clones of a "candidate gene" were picked up and a second round of SSCP confirmed the similarity of conformation of the inserts. Subsequently, a clone among the similar cDNAs conformations was chosen and sequenced.

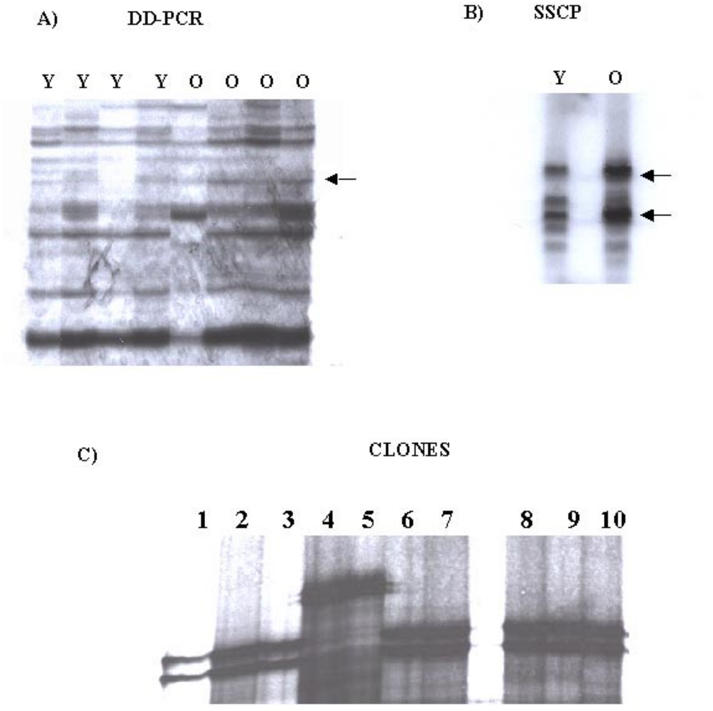

**FIGURE 3.** Eliminating false positives. (A) DD-PCR autoradiography of the samples analyzed. An area of the gel containing a more prominent band was cut as well as an equivalent area of less prominent band. Ten cycles of radiolabeled PCR were carried out using the cDNA extracted from these two areas. (B) Autoradiography of the SSCP gel shows the two cDNA strands, which are more prominent in the PCR amplification. The more prominent cDNA strand was extracted from the SSCP gel and was then amplified by another ten cycles of PCR and cloned into a TA-cloning plasmid. (C) Analyses of the clones revealed that the majority of them had the same DNA conformation. Therefore, one of these clones was sequenced and used as a probe for the Northern blot, which confirmed the DD-PCR pattern.

## MICROARRAYS

Recently the development of DNA microarrays (DNA chips) has provided a scale-up in hybridization assay technology because of their huge capacity for miniaturization and automation[26,27,28]. Although it is reminiscent of the filter-based arrays, microarray construction involves quite different procedures. The surface involved is glass rather than porous membranes; in addition, microarrays can be divided into two main classes according to their method of construction. One method of construction is a microarray of DNA clones delivered by microspotting. The DNA clones are prepared in advance and then printed onto the surface of a microscope slide. The second main class of construction is when a microarray of oligonucleotides is synthesized *in situ*. This approach has been pioneered by Affymetrix, Inc. (Santa Clara, CA) and typically involves a combination of photolithography technology from the semiconductor industry with the chemistry of oligonucleotide synthesis. As in the case of reverse dot-blotting, the DNA microarray technologies employ a reverse nucleic acid hybridization approach: the cDNA (probe) of interest is fixed to a solid support and the target is labeled in solution. Although the technology for establishing DNA microarrays was only developed in the last few years, there have been already numerous important applications and their impact on future medical research and diagnostic approaches is expected to be profound[26].

Lee et al.[29] have used microarray technology to examine the molecular events associated with aging in mammals by analyzing gastrocnemius muscle from young and old mice. Of the 6,347 genes surveyed in the oligonucleotide microarray, only 58 (0.9%) displayed a greater than twofold increase in expression levels as a function of age, whereas 55 (0.9%) displayed greater than twofold decrease in expression. These findings are in agreement with differential display analyses of gene expression in tissues of aging mice[18].

Microarray technology was also used to gain insight into the mechanisms that control lifespan and age-related phenotypes[30]. Dermal human fibroblast cell lines from normal young, normal middle-aged, and normal old and Hutchinson-Gilford progeria were analyzed. Messenger RNA levels were analyzed with high-density oligonucleotide arrays containing probes for more than 6,000 known human genes. Expression patterns of several cell lines were compared with the normal young cell line. They found that 61 genes (i.e., approximately 1% of the monitored genes) showed consistent expression level changes of more than twofold between young and middle age. In addition, the identified genes were grouped according to their function (cell cycle control proteins, chromosomal processing and assembly, fatty acids synthesis, extracellular matrix, DNA/RNA synthesis and repair, growth factors, tumor suppressors, and protein synthesis/processing). Recently, Park et al. compared the gene profile of human fibroblasts from progeria and elderly donors[31]. They observed that there was a closer relationship in gene expression between fibroblasts from progeria and old individuals, but not between replicative senescent fibroblasts and either of the two other models.

In conclusion, this technology has many foreseeable aging-related applications, which encompass a broad spectrum from basic research to clinical applications. Briefly, these include: (1) analyses of expression profiles of young and senescent cells, (2) identification of aging and longevity genes, and (3) auxiliary technique in the diagnoses of general disorders[28]. However, although the technology is already well established and offers numerous aging-related applications, it still remains expensive for routine applications. Furthermore, it suffers from a lack of standardized bioinformatic tools to analyze the large amount of data generated. As soon as these drawbacks are resolved, DNA microarrays can be widely used with several different applications. Consequently, we should expect an increase number of reports in the aging field using this technology.

## CONCLUSION

As the human genome project nears completion, the number of known genes is increasing rapidly. Of the approximate 30,000 genes to be accounted for, about 1% is thought to be related to aging; a great percentage of them remain to be identified and characterized. However, the future major task in the aging field will be to understand the function of the cloned genes, as well as to study the structure and the interactions of their protein products. The combination of the existing knowledge of genes undergoing expression changes in *in vivo* and in *in vitro* models of human aging and the application of advanced protein-related technologies, such as proteomics and phage display, will allow us to identify new proteins that associate with and regulate aging. This approach will improve the understanding of functional networks of aging genes and their proteins, which in turn will facilitate the understanding of the biology of normal aging and age-related disorders.

## ACKNOWLEDGMENTS

Part of the authors' experimental work presented in this mini-review article was supported by a European Union Biomed-2 "Genage" Grant BMH4-CT98-3149. We thank BD Biosciences CLONTECH for allowing us to use the schematic diagram of the SSH as Fig. 1 in this review.
